# Comparison of the virulence of three H3N2 canine influenza virus isolates from Korea and China in mouse and Guinea pig models

**DOI:** 10.1186/s12917-018-1469-1

**Published:** 2018-05-02

**Authors:** Xing Xie, Woonsung Na, Aram Kang, Minjoo Yeom, Heejun Yuk, Hyoungjoon Moon, Sung-jae Kim, Hyun-Woo Kim, Jeong-Ki Kim, Maoda Pang, Yongshan Wang, Yongjie Liu, Daesub Song

**Affiliations:** 1 Joint International Research Laboratory of Animal Health and Food Safety, College of Veterinary Medicine, Nanjing Agricultural University, Nanjing, 210095 China; 2 College of Pharmacy, Korea University, Sejong, 339-700 South Korea; 3 Institute of Veterinary Medicine, Jiangsu Academy of Agricultural Sciences, Key Laboratory of Veterinary Biological Engineering and Technology, Ministry of Agriculture, No.50 Zhongling Street, Nanjing, 210014 China; 4 Research Unit, Green Cross Veterinary Products, Yong-in, 17066 South Korea; 5 Department of Veterinary Medicine, Virology Lab, College of Veterinary Medicine, and School of Agricultural Biotechnology, BK21 Program for Veterinary Science, Seoul National University, Kwanak-gu, Seoul, 08826 South Korea; 6 Department of Veterinary Pathology, Small Animal Tumor Diagnostic Center, College of Veterinary Medicine, Konkuk University, 120 Neundong-ro, Seoul, 143-701 South Korea; 7 Institute of Food Safety and Nutrition, Jiangsu Academy of Agricultural Sciences, No.50 Zhongling Street, Nanjing, 210014 China

**Keywords:** H3N2 canine influenza virus, Pathogenicity, Transmissibility, BALB/c mice, Guinea pigs

## Abstract

**Background:**

Avian-origin H3N2 canine influenza virus (CIV) has been the most common subtype in Korea and China since 2007. Here, we compared the pathogenicity and transmissibility of three H3N2 CIV strains [Chinese CIV (JS/10), Korean CIV (KR/07), and Korean recombinant CIV between the classic H3N2 CIV and the pandemic H1N1 virus (MV/12)] in BALB/c mouse and guinea pig models. The pandemic H1N1 (CA/09) strain served as the control.

**Results:**

BALB/c mice infected with H1N1 had high mortality and obvious body weight loss, whereas no overt disease symptoms were observed in mice inoculated with H3N2 CIV strains. The viral titers were higher in the group MV/12 than those in groups JS/10 and KR/07, while the mice infected with JS/10 showed higher viral titers in all tissues (except for the lung) than the mice infected with KR/07. The data obtained in guinea pigs also demonstrated that group MV/12 presented the highest loads in most of the tissues, followed by group JS/10 and KR/07. Also, direct contact transmissions of all the three CIV strains could be observed in guinea pigs, and for the inoculated and the contact groups, the viral titer of group MV/12 and KR/07 was higher than that of group JS/10 in nasal swabs. These findings indicated that the matrix (M) gene obtained from the pandemic H1N1 may enhance viral replication of classic H3N2 CIV; JS/10 has stronger viral replication ability in tissues as compared to KR/07, whereas KR/07 infected guinea pigs have more viral shedding than JS/10 infected guinea pigs.

**Conclusions:**

There exists a discrepancy in pathobiology among CIV isolates. Reverse genetics regarding the genomes of CIV isolates will be helpful to further explain the virus characteristics.

## Background

Influenza A virus (IAV) is a highly contagious pathogen. The natural hosts of IAV are birds, but certain IAV lineages may infect additional mammalian hosts, especially humans, swine, and equines [1, 2]. Dogs were not considered a reservoir species for influenza virus, because no evidence of the continuous spread of IAV among dogs was available until 2004, when an H3N8 influenza virus of equine origin caused an extensive epizootic of respiratory disease in racing dogs in Florida [2]. In 2007, another canine influenza outbreak was confirmed in South Korea [3]; sequence analysis revealed that the causal agent was an avian-origin H3N2 influenza virus, which was then demonstrated to be capable of direct transmission between dogs [4]. Outbreaks of infections caused by avian-origin H3N2 canine influenza virus (CIV) have been continuously reported in South Korea [4, 5], China [6, 7] and Thailand [8] since 2007, and avian-origin H3N2 CIV has become the most prevalent subtype in Asia [1].

Recently, a large number of studies has evaluated the pathogenicity of H3N2 CIV [7, 9–11]. From 2009 to 2010, Lin et al. (2012) isolated six strains of avian-origin H3N2 CIVs in Jiangsu Province of China, and molecular analysis indicated that all eight genes of the six strains shared high sequence identity (> 99%) with the H3N2 CIV strain isolated in South Korea. The pathogenicity of the representative Chinese H3N2 CIV strain A/canine/Jiangsu/06/2010 (JS/10) has been characterized in both mice [7, 10] and dogs [12]. Previous studies also characterized and compared the pathogenicity of the classical Korean H3N2 CIV A/canine/Korea/01/2007 (KR/07) in various animal models, including mice [5], guinea pigs [13] and dogs [9]. In 2012, an H3N2 CIV reassortant (A/canine/Korea/MV1/2012, MV/12), the M gene from the pH1N1 influenza virus and seven other genes from classic H3N2 CIVs, was isolated from a sick dog in South Korea [11, 14]. The infection dynamics of this reassortant strain were investigated via experimental infection in dogs and ferrets [14]. Nevertheless, the pathogenicity and transmissibility of these CIV isolates have not been compared and analyzed under the same experimental conditions to date.

The epidemic spread of H3N2 avian-origin CIVs represents not only highly contagious pathogens for dogs but also a public health concern. Because dogs are the most intimate companions of humans, the close contact between humans and dogs may increase the potential for the transmission of influenza viruses to humans [15–17]. Companion animals in South Korea and China have lots of opportunities (i.e., international travel and trade) to encounter H3N2 CIV-infected dogs in pet shops, veterinary clinics or outdoor areas, owing to the endemicity of the virus in both countries recently [10, 15]. Therefore, a comparison of the pathogenicity and transmissibility of different CIV strains has special and important meaning for both countries.

Mice have shown promising potential for underlying the basic viral pathogenesis of influenza virus, which have traditionally been used as a mammalian animal model [5, 18, 19]. Alternatively, guinea pigs have also been reported to be a relevant model of influenza virus infection and are suitable for evaluations of the transmissibility of IAVs in mammalian hosts [18, 20]. Here, in order to compare the pathogenicity and transmissibility of different CIV strains, investigations were conducted with three H3N2 viruses (Chinese CIV JS/10, Korean CIV KR/07 and reassortant CIV MV/12) and one pandemic H1N1 CA/09 strain using mouse and guinea pig models under the same experimental conditions.

## Methods

### Experimental animals

One hundred six-week-old specific pathogen-free (SPF) female BALB/c mice (18–20 g) and 75 six-week-old SPF female outbred Hartley guinea pigs (300–350 g body weight) were purchased from Yangsung Laboratory Animal (Yangsung, South Korea). All experiments with animals and viruses were conducted in biosafety level 2-plus facilities at Korea University.

### Ethics statements

Veterinarians took the samples for analysis purposes and to check the health status of the mice and guinea pig population. Before conducting the study, approval for conducting the animal experiments was obtained from the Animal Ethics Committee of Korea University, with the approval number of KUIACUC-2016-132.

### Viruses

Three virus strains of the avian-origin H3N2 subtype [Chinese CIV strain A/canine/Jiangsu/06/2010 (JS/10), Korean CIV strain A/canine/Korea/01/2007 (KR/07), and H3N2 CIV reassortant A/canine/Korea/MV1/2012 (MV/12)] and one pandemic H1N1 influenza virus [A/California/04/2009 (CA/09)] were used in this study. Four virus strains of the second passage were propagated in 10-day-old SPF embryonated chicken eggs. No sequence differences were found between the original wild viruses and the egg adapted viruses. Viral titers were measured by calculating the 50% egg infectious dose (EID_50_/mL) of the viral stock by using the method of Reed and Muench [21]. The titers of the four viral strains (JS/10, KR/07, MV/12 and CA/09) were 10^6.83^ EID_50_/mL, 10^7.50^ EID_50_/mL, 10^8.17^ EID_50_/mL and 10^8.17^ EID_50_/mL, respectively.

### Mouse infections

To compare the virulence of JS/10, KR/07, MV/12 and CA/09, mice infected with each virus were set as a separate experimental group. Mice inoculated with CA/09 and phosphate-buffered saline (PBS) were used as the positive and negative controls, respectively. All of the mice in different groups were housed in individual compartments in stainless-steel wire cages [22]. For each virus group, 15 mice were anesthetized by intramuscular injection of Zoletil (15 mg/kg, Virbac, Carros, France) in 0.1 mL of PBS, and then inoculated with the virus. For intranasal inoculation, 10^6^ EID_50_/mL of each virus in 50 μL of PBS was administered into the nostrils of the anesthetized mice. The inoculated mice in each group were distinguished by the ear tags. Three of the mice in inoculated group were euthanized by carbon dioxide (CO_2_) inhalation, and then sampled for virus load titration of different organs, including the brain, heart, liver, lung, spleen, kidney, intestine and feces, for each virus at 1, 4, 7, 11 and 14 days post-inoculation (dpi). Three mice of each time point were inoculated with PBS as a negative control. Beddings were changed every three days before mice were killed humanely at indicated time points. Additionally, five mice in each virus group and PBS group were selected to monitor their clinical signs, survival rates and body weight loss for 14 consecutive days. The observers were blinded as to the experimental treatments and they had veterinary medical qualifications to make assessments about clinical signs [23]. Mice were euthanized for humane reasons when they lost more than 25% of their original body weight [18].

### Guinea pig infections

To compare virulence and to assess the efficiency of the transmission of each virus by direct contact, 72 guinea pigs were randomly divided into four virus experimental groups; the remaining three guinea pigs were inoculated with PBS as a negative control. Nine of 18 guinea pigs of each group were randomly selected and put in one cage to be anesthetized. Anaesthesia was induced by intramuscular injection of Zoletil (20 mg/kg, Virbac, Carros, France) in 0.1 mL of PBS. Then intranasal administration of 10^6^ EID_50_/mL of JS/10, KR/07, MV/12 or CA/09 in a total volume of 300 μL (150 μL per side) into the nostrils of every guinea pig was performed. Guinea pigs in the control PBS group were inoculated with the same volume of PBS. Another nine guinea pigs of each virus groups and the three guinea pigs in the PBS group were housed in separate cages in independent isolators. The ambient conditions were set at air temperature of 22 °C with a relative humidity of 30% [22, 23]. The heads of three of the nine guinea pigs in each virus-inoculated group were stained with crystal violet for nasal swab collection according to a pre-designated schedule.

Twenty-four hours later, nine naive guinea pigs were introduced into each virus group as the contact group. By then, each cage contained only one infected and one naive contact guinea pig. Tails of three of the nine guinea pigs in each virus-contact group were stained with crystal violet. Nasal swabs for viral titration were collected from each guinea pig stained with crystal violet in the inoculated groups, contact experimental groups and PBS control group every day until 10 dpi by applying moistened cotton wads to both nostrils. Guinea pigs in the PBS and contact groups were handled first to prevent inadvertent physical transmission of the virus by the researchers. In addition, all materials used to handle and manipulate the animals during nasal wash collection were changed between guinea pigs in different virus groups [24]. Three guinea pigs in each virus-inoculated group at 3 and 5 dpi and three contact guinea pigs in the four virus experimental groups at 3 and 5 days post-exposure (dpe) were euthanized by CO_2_ inhalation. The organs were collected from the guinea pigs, including the lung, trachea, nasal turbinate, soft palate, brain, and rectum. Whole lung tissues connecting the tracheas were collected, from both the inoculated and contact guinea pigs of the four virus groups for the gross lesion observation. (-I) represents for the viral inoculation group and (-C) represents for the virus contact group of four viruses.

### Virus titration and serological test

Nasal viral shedding and the viral loads of all organs collected from both the mice and the guinea pigs were quantified by real-time PCR as described previously [7, 10]. In brief, the amount of RNA of three avian-origin CIVs and human-origin influenza viruses was calculated from the standard curve on Step One plus Real Time PCR System. Blood samples collected from infraorbital veins in BALB/c mice and hearts in guinea pigs from all groups were used for serological assessment prior to infection and at 10 dpi. Sera from the experimentally inoculated groups were two fold serially diluted in duplicate wells with an initial dilution of 1:10 and the antibodies against CIV were measured using a hemagglutination inhibition (HI) assay [25, 26] coupled with a commercially available competitive nucleoprotein (NP) ELISA kit (Bionote, Hwaseong-si, South Korea) [27]. HI titers were expressed as the inverse of the highest dilution that yielded complete inhibition of haemagglutination activity.

### Histopathological examination

To evaluate the histopathology of the lung tissues, necropsies were conducted following standard procedures. All mice and guinea pigs were humanly euthanized by inhalation of carbon dioxide in a gas chamber. Briefly, the mice were sacrificed at 7 dpi, and lung tissues from the guinea pigs in the four virus groups were collected for pathological examination at 3 dpi and 5 dpe, respectively. Four micrometer-thick sections were prepared from the paraffin-embedded tissues by immersing the lung tissues fixed in 10% neutral buffered formalin. Sections were stained with hematoxylin and eosin (H&E) as previously described [10]. Histopathological lesions of each lung tissue sample from the guinea pigs were evaluated in two categories representing pneumonic lesions (lymphocyte infiltration and congestion or hemorrhaging). Each category was graded as 0 (normal), 1 (mild), 2 (moderate) and 3 (severe) depending on the lesion severity [7].

### Statistical analysis

Data were collected and analyzed by using MS Excel 2010 and the SPSS Statics v20.0 software. Body weight loss and viral titers were analyzed by using analysis of variance (ANOVA) followed by Tukey’s multiple comparison test, with *P* < 0.05 or *P* < 0.01 considered a significant difference.

## Results

### Clinical signs and body weight changes in the mouse model

Mice infected with CA/09 showed progressive clinical signs, such as decreased activity, labored breathing, lack of appetite, and ruffled fur. All of these mice died after 7 dpi. In contrast, all of the mice inoculated with the three H3N2 CIV strains and PBS survived and demonstrated no obvious clinical signs.

**Fig. 1:**
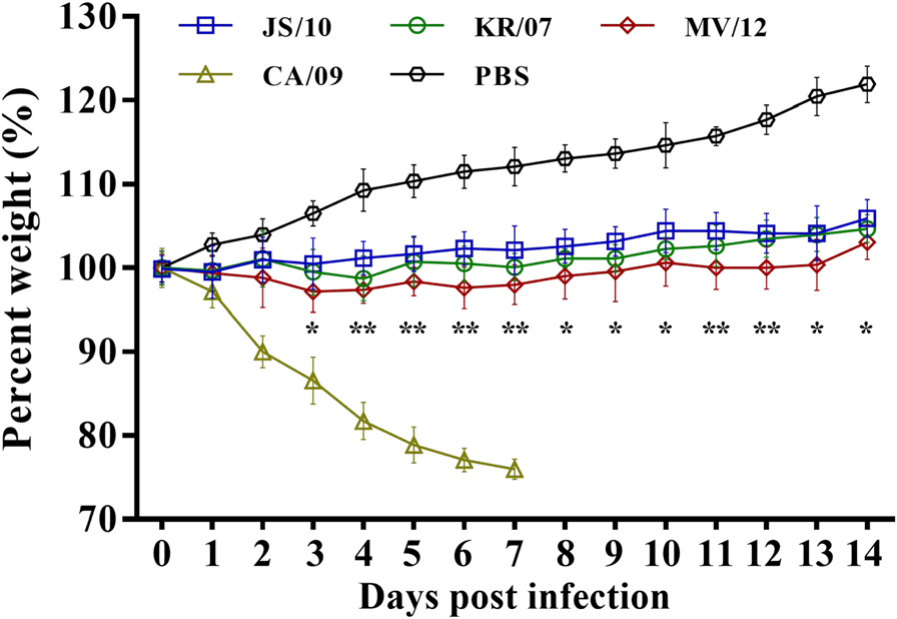
Body weight changes in mice inoculated with four influenza virus strains. Four experimental groups of 6-week-old BALB/c mice were challenged with 10^6^ EID_50_/mL of the JS/10, KR/07, MV/12 and CA/09 strains. Mice inoculated with same volume of PBS served as the negative control. Mice were monitored for body weight loss throughout the observation period for 14 days. Each error bar indicates the standard deviation. The results are expressed in terms of percent body weight. *, *P* < 0.05, or **, *P* < 0.01, indicates significantly different weight compared between group JS/10 and MV/12

As depicted in Fig. 1, the body weights of the mice inoculated with PBS gradually increased, with the average body weight increasing by more than 20% until 14 dpi. The body weights of the mice in three H3N2 CIV groups demonstrated similar trends. No significant differences were found in body weight between the JS/10 and KR/07 groups or between the KR/07 and MV/12 groups. However, the body weights of the mice in the MV/12 group were significantly decreased compared with those of the mice in the JS/10 group from 3 to 14 dpi (*P* < 0.05), especially from 4 to 7 and 11 and 12 dpi, when the body weights of the mice in the MV/12 group were significantly decreased compared to those of the mice in the JS/10 group (*P* < 0.01). In contrast, an average weight loss of more than 15% was observed in the mice inoculated with CA/09 by 3 dpi, subsequently a rapid and significant weight loss of up to 25% of the body weight until 7 dpi, when all mice were euthanized.

### Quantitation of the viral RNA loads in the mouse model

**Fig. 2:**
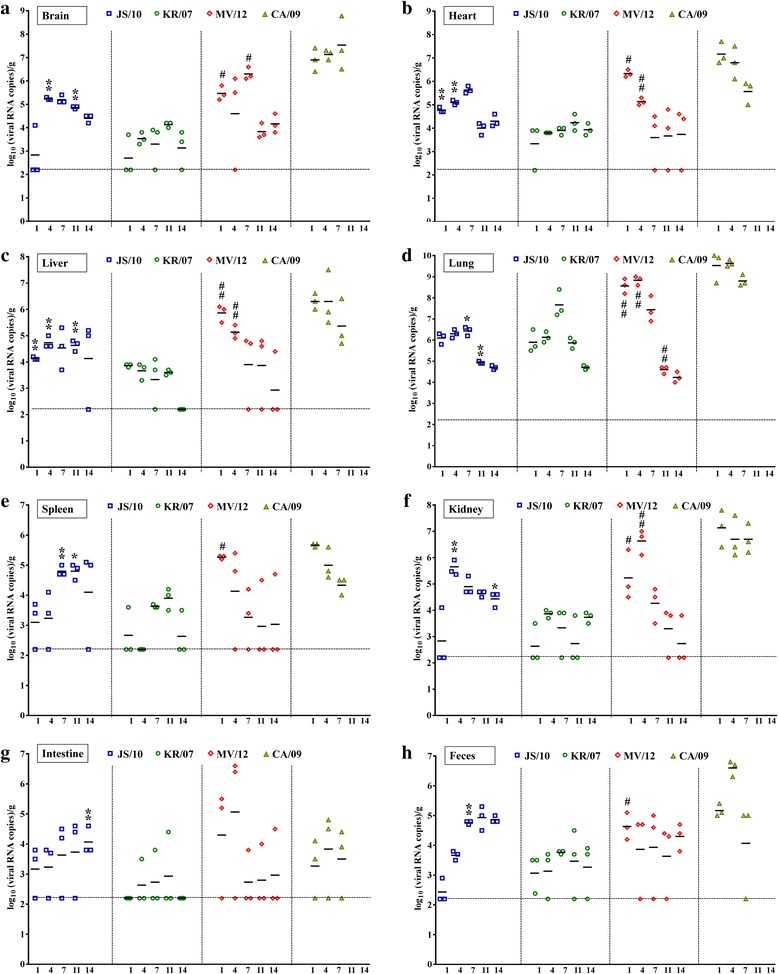
Viral loads in collected tissues and fecal samples from mice at five different time points after infection with four virus strains. Mice were inoculated with 10^6^ EID_50_/mL of the JS/10, KR/07, MV/12 and CA/09 strains. In each virus group, the brain (**a**), heart (**b**), liver (**c**), lung (**d**), spleen (**e**), kidney (**f**), intestine (**g**) and feces (**h**) were collected from the mice to determine the viral loads using real-time PCR at 1, 4, 7, 11 and 14 days post-challenge. *, *P* < 0.05, or **, *P* < 0.01, indicates significantly different virus titers compared between group JS/10 and KR/07. #, *P* < 0.05, or ##, *P* < 0.01, indicates significantly different virus titers compared between group MV/12 and KR/07. For viral loads in different organs mentioned above, the results are expressed as log_10_ (viral RNA copies)/g. The horizontal line means the detection limit of this assay (158 copies of RNA per g)

Real-time PCR was used to assess the kinetics of the viral RNA loads in organs including the brain, heart, liver, lung, spleen, kidney, intestine and the feces of the inoculated mice with the four viruses. All the three H3N2 CIVs and pandemic H1N1 virus RNA could be detected by the quantitative PCR. For the inoculated mice in the CA/09 group, only the viral RNA loads at the first three time points were tested, since all mice were euthanized after 7 dpi. Importantly, we observed that the viral titers were highest in the lung tissues and lowest in the intestinal tissues. Moreover, the highest viral titers were found in the mice in group CA/09, whereas group KR/07 had the lowest viral titers with the exception of the lung tissues (Fig. 2). The dynamic changes of viral titers in the tissues and feces of the mice in the JS/10 and KR/07 groups were similar, with peak viral titers were observed at 4, 7 or 11 days after infection, followed by a decline at 14 dpi. However, in contrast to the JS/10 and KR/07 viruses, the peak viral titers of MV/12 in the mouse organs (except for the brain) and feces were observed at the earliest two time points (1 or 4 dpi). The viral titers in group CA/09 also reached the peak at 1 or 4 dpi in all organs and the feces except for the brain and intestine. The viral titers in the different organs (except for the lung and intestine) and the feces were significantly higher in the mice infected with JS/10 than in the mice infected with KR/07 at the different post-infection time points.

To be noted, the lung was the main target organ, because viral RNA could be detected in the lung from all the virus inoculated groups at each time point and the titers were higher compared to the other tissues. As depicted in Fig. 2d, the viral titers of groups MV/12 and CA/09 reached the peak at 4 dpi, whereas those of groups JS/10 and KR/07 reached the peak at 7 dpi. The peak viral titers of group MV/12 were significantly higher than those of groups JS/10 and KR/07 respectively. However, with the prolongation of viral infection, the viral titers of group MV/12 became significantly lower compared to group KR/07 at 11 dpi. Additionally, the viral titers in the lung tissues were significantly lower in group JS/10 than those in group KR/07 at 7 and 11 dpi.

### Histopathological findings in the mouse lungs

**Fig. 3:**
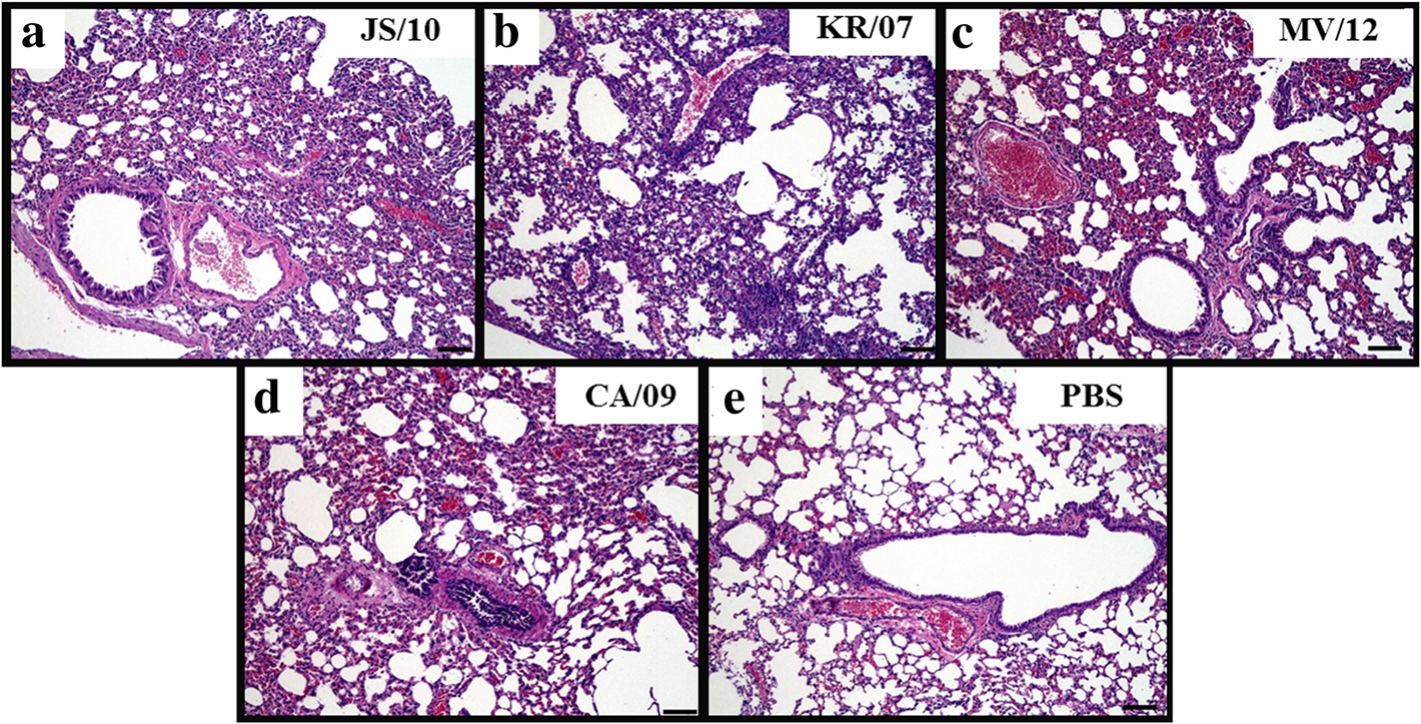
Histopathological lesions in lung samples from mice infected with the four virus strains at 7 dpi. Histopathological findings in the lungs of mice at 7 days post-inoculation with 10^6^ EID_50_/mL of the JS/10, KR/07, MV/12 and CA/09 strains. All inoculated groups demonstrated histopathological pneumonic lesions. (A) – (E) are representative microscopic images of the histopathological pneumonic lesions from each group (X100). (A) JS/10 and (B) KR/07 resulted in mild lymphocyte infiltration and congestion. (C) MV/12 and (D) CA/09 resulted in mild lymphocyte infiltration and moderate congestion and hemorrhaging. (E) Lung tissue in the normal state

To compare the pathological findings in mice infected with different viruses, the lung tissues from each group at 7 dpi were selected to perform a histopathological analysis, because the viral titers of the lung tissues were the highest among all tissues. All of the sampled tissues from the mice in the four virus-infected groups showed lesions to different extents. The lung tissues of the mice infected with JS/10 (Fig. 3a) and KR/07 (Fig. 3b) showed mild histopathological lesions with widened lung interstitial spaces, narrowed bronchial lumens, mild infiltration with a number of inflammatory cells and thickening in the alveolar septum. In contrast, the mice infected with MV/12 (Fig. 3c) and CA/09 (Fig. 3d) showed moderate lymphocyte infiltration and congestion or hemorrhage. No histopathological lesions were observed in the lung tissues from the PBS group (Fig. 3e).

### Influenza virus strain transmission among Guinea pigs by direct contact

**Fig. 4:**
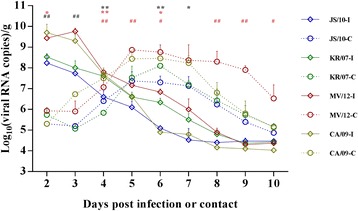
Nasal swab shedding of guinea pigs infected with the four virus strains in both the inoculation and contact groups. Guinea pigs were inoculated with the JS/10, KR/07, MV/12 and CA/09 virus strains. After 24 h on 1 dpi, additional naive guinea pigs were placed into each virus group as the contact group. Nasal swabs were collected every day for determination of the viral loads using real-time PCR and the results are expressed as log_10_ (viral RNA copies)/g. The solid line represents for the viral inoculation group (-I) and the dotted line represents for the virus contact group (-C) of four viruses. Each error bar indicates the standard deviation. *, *P* < 0.05, or **, *P* < 0.01, indicates significantly different virus titers in nasal swabs compared between group JS/10 and KR/07. * in black, represents significant difference in inoculation group, while * in red demonstrates significant difference in contact group. #, *P* < 0.05, or ##, *P* < 0.01, indicates a significant difference in virus titers for group MV/12 compared with group KR/07. # in black, represents significant difference in inoculation group, while # in red demonstrates significant difference in contact group. All significant differences are shown above the figure

To compare the pathogenicity of the four influenza virus strains and to evaluate the capacity of the four viruses to be transmitted between guinea pigs by direct contact, nasal washes from both the inoculated and contact guinea pigs were collected to test the presence of the virus. As shown in Fig. 4, the nasal swab viral titers from guinea pigs in each group showed similar trends after infection by inoculation or contact. In the inoculated group, the viral titers of each virus group reached peak levels at 2 or 3 days and then declined to the lowest levels at 8, 9 or 10 days. The peak viral titers of the JS/10, KR/07, MV/12 and CA/09 groups were 10 ^8.23^, 10^8.53^, 10^9.77^ and 10^9.70^ copies/g, respectively. In contrast, for the direct contact guinea pigs, the viral titers of each group first had lower levels of approximately 10^5.50^ copies/g and then gradually increased to the peak values at 5 to 6 dpi (4 to 5 dpe). In the contact groups, the peak viral titers of the JS/10, KR/07, MV/12 and CA/09 groups were 10^7.37^, 10^8.10^, 10^8.87^ and 10^8.47^ copies/g, respectively, which were lower than those of the inoculated groups.

For the inoculated group, the viral titers of group CA/09 were highest between 3 to 8 dpi, whereas the titers of group JS/10 were lowest between 2 to 5 dpi. The viral titers of group JS/10 were significantly lower than those of group KR/07 at 4, 6 and 7 dpi, and the viral titers of group MV/12 group were significantly higher than those of group KR/07 at 2 and 3 dpi. Additionally, no significant differences were found between groups JS/10 and KR/07 or between groups KR/07 and MV/12 at 8 to 10 dpi. For the contact group, the viral titers of groups CA/09 and MV/12 were higher than those of groups JS/10 and KR/07 except for 2 dpi. The viral titers of group JS/10 were significantly lower than those of group KR/07 at 2 and 6 dpi, whereas the viral titers of group JS/10 were significantly higher than those of group KR/07 at 4 dpi. Additionally, the viral titers of group MV/12 were significantly higher than those of group KR/07 at 4, 5, 7, 8, 9 and 10 dpi.

### Serological analysis of Guinea pigs both by inoculation and direct contact

Seroconversion was confirmed by nucleoprotein-specific ELISA and a HI assay. Seroconversion was observed in all guinea pigs regardless of whether they were infected by inoculation or direct contact (shown in Table 1). The average titers of groups MV/12 and CA/09 were significantly higher than those of groups JS/10 and KR/07. Additionally, the HI titers of guinea pigs infected through inoculation were higher than those of guinea pigs infected by direct contact.

**Table 1:** Serological responses of guinea pigs against four virus strains in both inoculated and contact groups

Virus group	Positive rate of NP		Average HI titer^*^	
	Day 0	Day 10	Day0	Day 10
JS/10 inoculation	0/3	3/3	< 10	53.3
JS/10 contact	0/3	3/3	< 10	26.7
KR/07 inoculation	0/3	3/3	< 10	66.7
KR/07 contact	0/3	3/3	< 10	26.7
MV/12 inoculation	0/3	3/3	< 10	133.3
MV/12 contact	0/3	3/3	< 10	80.0
CA/09 inoculation	0/3	3/3	< 10	266.7
CA/09 contact	0/3	3/3	< 10	106.7

^*^Samples with an HI titer < 10 were classified as negative

### Quantitation of the viral RNA loads in the Guinea pig model

**Fig. 5:**
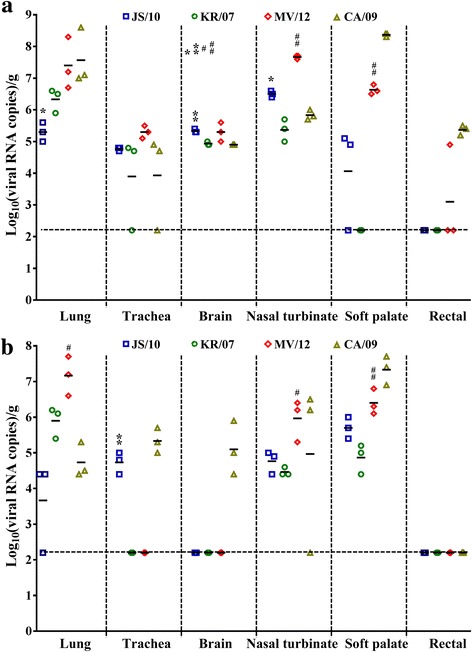
Viral loads in tissues collected from guinea pigs infected with the four virus strains at 3 dpi for the inoculated group and 5 dpe for the contact group. Guinea pigs were inoculated with 10^6^ EID_50_/mL of the JS/10, KR/07, MV/12 and CA/09 strains. Organs including the lung, trachea, brain, nasal turbinate, soft palate and rectal were collected for the determination of the viral loads using real-time PCR at 3 dpi and 5 dpe, for the inoculated group (**a**) and contact group (**b**) for each virus, respectively. The results are expressed as log_10_ (viral RNA copies)/g. *, *P* < 0.05, or **, *P* < 0.01, indicates significantly different virus titers compared between JS/10 and KR/07 virus group. #, *P* < 0.05, or ##, *P* < 0.01, indicates a significant difference in virus load for the group MV/12 compared with group KR/07. For viral loads in different organs mentioned above, the results are expressed in terms of mean virus titer logEID_50_. The horizontal line means the detection limit of this assay (158 copies of RNA per g)

According to the nasal swab viral titers discussed above, the lung, trachea, brain, nasal turbinate, soft palate and rectum of the guinea pigs in the inoculated and contact groups were collected to determine the viral loads at 3 and 6 dpi, respectively. As depicted in Fig. 5a, the viral titers of all tissues except for the brain were higher in group MV/12 than in groups JS/10 and KR/07, whereas the viral titers were higher in the trachea, brain, nasal turbinate and soft palate in group JS/10 than in group KR/07. We also observed that the viral titers of the nasal turbinate were highest in all tissues for groups JS/10 and MV/12, whereas the highest viral titers for groups KR/07 and CA/09 were found in the lung and soft palate, respectively.

For the contact group (shown in Fig. 5b), the viral titers of the lung, nasal turbinate and soft palate were significantly higher for group MV/12 than for group KR/07, whereas the viral titers of the trachea and soft palate were significantly higher for group JS/10 than for group KR/07. However, we observed that the viral titers of the lung tissues were significantly higher for group KR/07 than for group JS/10. The viral titers in the lung tissues were the highest for groups KR/07 and MV/12, whereas the viral titers of the soft palate were the highest for groups JS/10 and CA/09. Additionally, none of the four viruses were detected in the rectums of any of the guinea pigs.

### Gross lesions and histopathological findings in the Guinea pig lungs

**Fig. 6:**
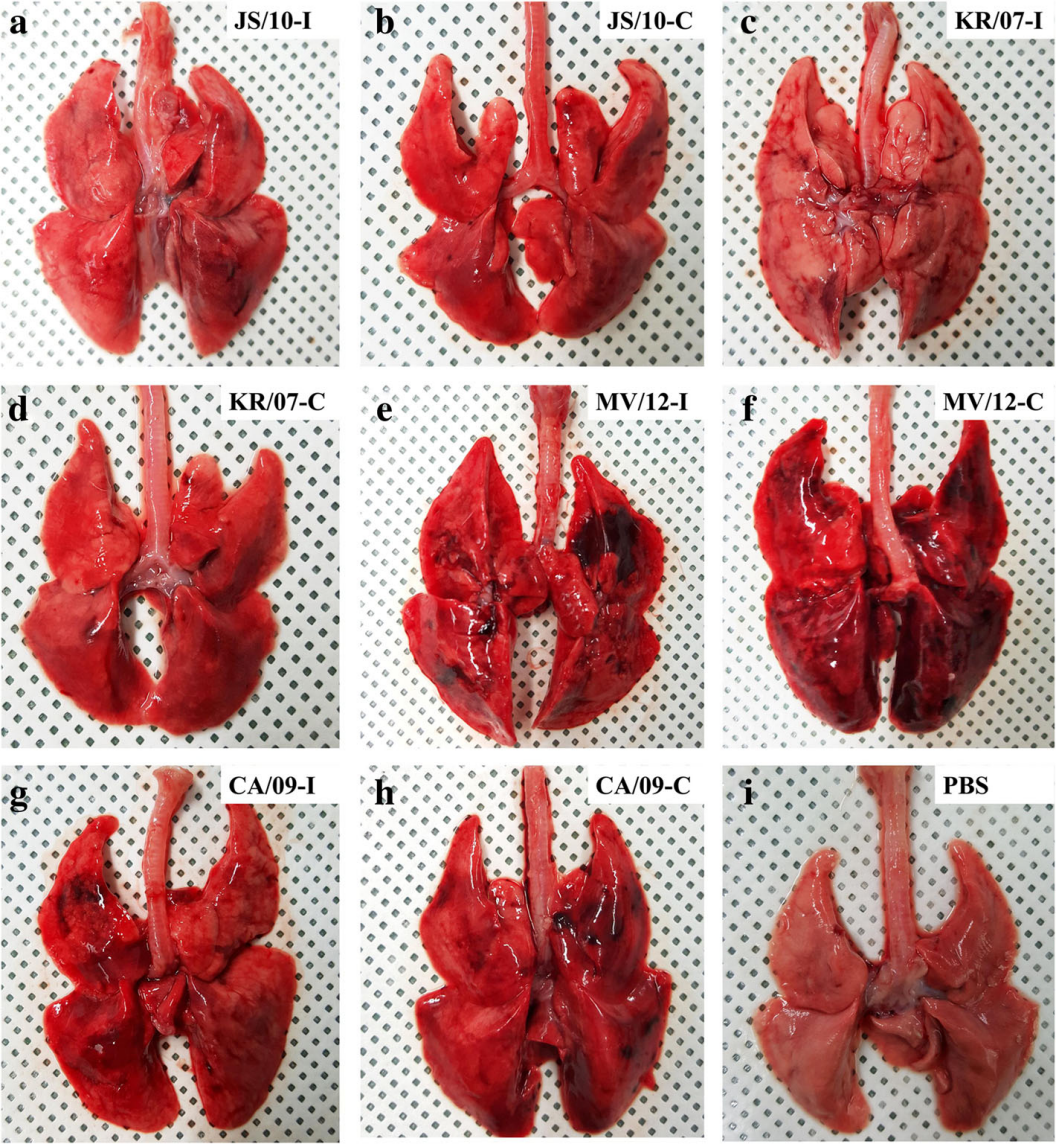
Gross lesions of lung samples from guinea pigs infected with the four virus strains in both the inoculation and contact groups. Guinea pigs were inoculated with 10^6^ EID_50_/mL of the JS/10, KR/07, MV/12 and CA/09 strains. After 24 h on 1 dpi, additional naïve guinea pigs were placed into each virus group as the contact group. Pictures were taken of the gross lesions of the viral inoculation groups at 3 dpi for JS/10 (**a**), KR/07 (**c**), MV/12 (**e**) and CA/09 (G) and of the virus contact groups at 6 dpi (5 dpe) for JS/10 (**b**), KR/07 (**d**), MV/12 (**f**) and CA/09 (H). Macroscopic images of guinea pig lungs in the PBS negative control (I) were also taken

According to the viral titers corresponding to nasal swab shedding by the guinea pigs in the four virus groups, the guinea pigs in the inoculated and contact groups were selected for sacrifice at 3 and 6 dpi, respectively. No apparent differences were observed for the guinea pigs in groups JS/10-I (Fig. 6a) and KR/07-I (Fig. 6c) infected through inoculation, and only moderate hemorrhaging and edema were observed in parts of the left or right caudal lobes. However, MV/12-I showed the most severe lesions; the left caudal and right cranial lobes of the infected lungs showed the most severe pneumonia, and a wide range (more than 25% of the lobes) of the lungs appeared to show hemorrhages, especially in the right upper lobe (Fig. 6e). While the range of hemorrhages and some parts of edema in the lungs in group CA/09-I (Fig. 6g) was less severe than that in group MV/12-I. In contrast, the lung tissues of the guinea pigs in the PBS group showed no pneumonia (Fig. 6i). Similar to the inoculated group, the lung tissues of the contact groups showed gross lesions to different degrees. Group MV/12-C also demonstrated the most severe gross lesions (Fig. 6f) with all four lung lobes almost full of hemorrhages (more than 80% of the lobes). The lung tissues in group CA/09-C showed reddish hemorrhages and a small amount of edema (Fig. 6h), whereas the lungs in groups JS/10-C (Fig. 6b) and KR/07-C (Fig. 6d) showed slight gross lesions with very little hemorrhaging.

**Fig. 7:**
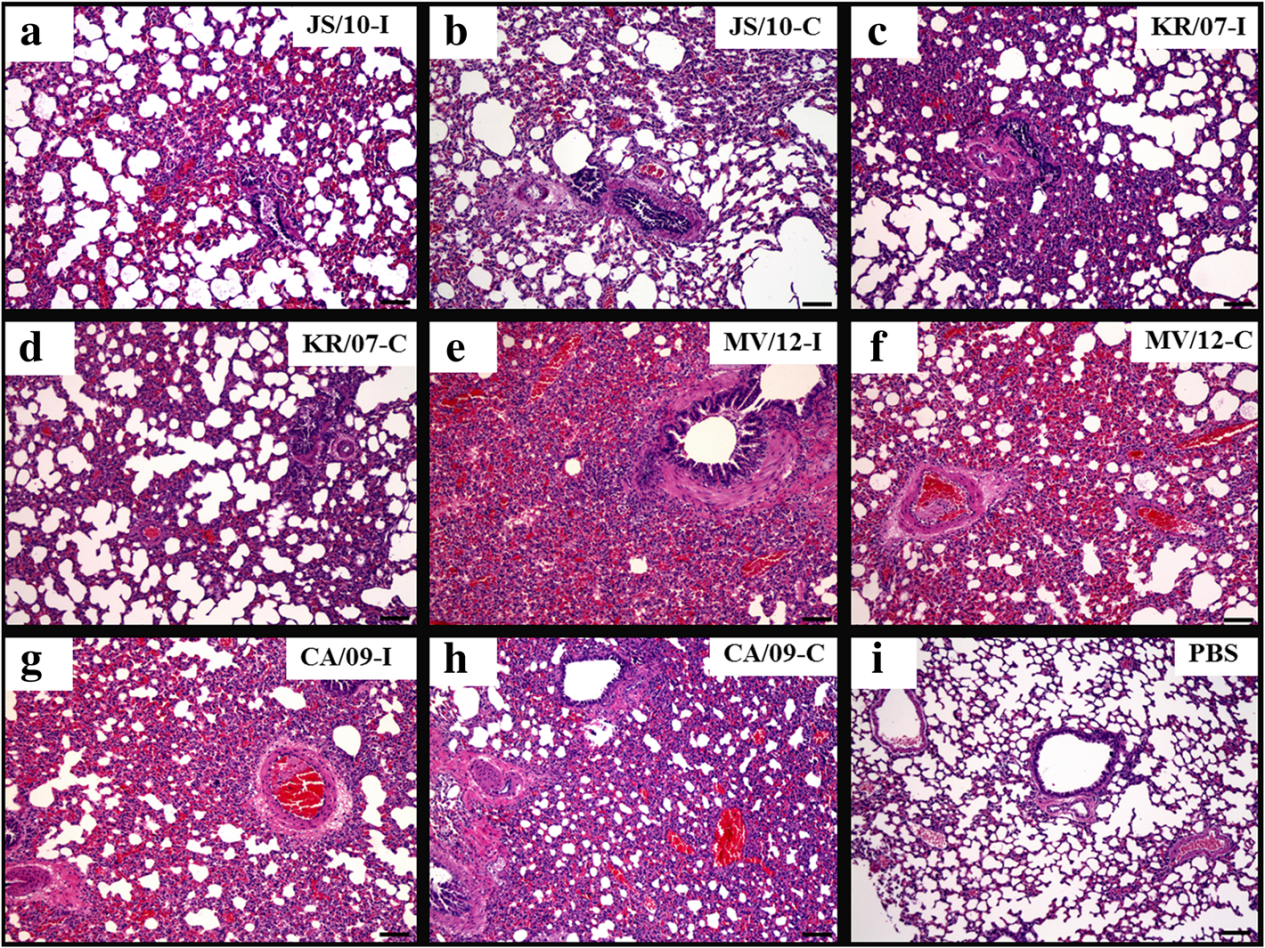
Histopathological lesions in guinea pig lung samples after infection with the four virus strains at 3 dpi for the inoculation group and 5 dpe for the contact group. Guinea pigs were inoculated with 10^6^ EID_50_/mL of the JS/10, KR/07, MV/12 and CA/09 strains. Each microscopic image represents histopathological pneumonic lesions in the viral inoculation group (-I) at 3 dpi for JS/10 (**a**), KR/07 (**c**), MV/12 (**e**) and CA/09 (**g**) and in the virus contact group (-C) at 6 dpi (5 dpe) for JS/10 (**b**), KR/07 (**d**), MV/12 (**f**) and CA/09 (**h**) (X100). The lesion scores of each image was as following: (**a**) LI: 1 and CH: 2; (**b**) LI: 1 and CH: 1; (**c**) LI: 1 and CH: 1;(**d**) LI: 1 and CH: 1; (**e**) LI: 3 and CH:3; (**f**) LI: 2 and CH: 3; (**g**) LI: 2 and CH: 2;(**h**) LI: 2 and CH: 1; (**i**) LI: 0 and CH: 0

As shown in Fig. 7, the pattern of the histopathological findings was consistent with the gross lesions described above. Among the inoculated groups, groups JS/10-I (Fig. 7a) and KR/07-I (Fig. 7c) showed mild to moderate histopathological lesions, with mild lymphocyte infiltration and slight to moderate hemorrhaging. While group MV/12-I (Fig. 7e) demonstrated the most severe histopathological lung lesions, with severe lymphocyte infiltration and congestion or hemorrhaging. The histopathological lesions of group MV/12-I were more severe than those of group CA/09-I (Fig. 7g), which showed moderate lymphocyte infiltration and congestion or hemorrhaging. Histopathological lesions were not observed in the lung tissues of guinea pigs in the PBS group (Fig. 7I). Additionally, the histopathological findings of the lungs in the contact groups were similar to those of the inoculated group. To compare the histopathological lesions more directly, the histopathological lesions were evaluated in two categories based on lymphocyte infiltration (LI), and congestion or hemorrhage (CH), which were graded as 0 (normal), 1 (mild), 2 (moderate) and 3 (severe). Therefore, the lesion scores of each image was as following: (A) LI: 1 and CH: 2; (B) LI: 1 and CH: 1; (C) LI: 1 and CH: 1; (D) LI: 1 and CH: 1; (E) LI: 3 and CH: 3; (F) LI: 2 and CH: 3; (G) LI: 2 and CH: 2; (H) LI: 2 and CH: 1; (I) LI: 0 and CH: 0.

## Discussion

Outbreaks of infections caused by H3N2 CIV, which can be transmitted directly in dogs, have been constantly reported in Asian countries since 2007, including South Korea, China, and Thailand [3, 7, 10, 11, 28]. Recently, Asian canine H3N2 virus was also imported to U.S. [29, 30]. In this study, we assessed the pathogenicity and transmissibility of classic H3N2 virus strains (Chinese CIV JS/10, Korean CIV KR/07 and reassortant CIV MV/12) under the same conditions. Because the pathogenesis of the pandemic H1N1 CA/09 strain has been well addressed in animal models, including mice [19, 31] and guinea pigs [32], we used this viral strain as a reference.

Body weight loss is the most common parameter used to assess influenza viral pathogenicity in mice [7, 33]. In this study, three H3N2 CIV strains showed similar trends in body weight changes, with a slight decrease at one to three days, followed by a slight increase until 14 dpi. These findings were consistent with the results from previous studies [5, 10, 34]. Regarding the individual viral strains, JS/10 resulted in lower weight loss than MV/12 from 3 to 14 dpi, but no significant difference was found between JS/10 and KR/07.The viral titer of group MV12 was significantly higher than that of groups JS/10 or KR/07 in almost all infected tissues, while group JS/10 showed significantly higher titer in most of the tissues than group KR/07, except for the lung. And the lung histopathological findings of the mice infected with the four viral strains were consistent with the body weight change and viral load trends. Therefore, the data obtained in the mouse model indicated that the pathogenicity of MV/12 was higher than the pathogenicity of JS/10 and KR/07.

The guinea pig model has been reported to offer advantages over other mammalian models for the study of influenza virus transmission [35]. In this study, viral RNA loads were detectable in the nasal swabs of all guinea pigs infected with the four viral strains regardless of whether the infection route was inoculated or direct contact. Notably, the transmissibility results in guinea pigs of this study was in conflict with a previous report conducted with the H3N2 CIV strain KR/07 [13], in which no direct contact transmission was observed in guinea pigs. The reason for the inconsistent results may partly be due to different initial co-caged time or detection limit for the viral titers. For the inoculated and the contact groups, the viral titers of nasal swabs in group MV/12 were higher than those of groups JS/10 and KR/07, and the viral titers of group KR/07 were higher than those of group JS/10 at most time points. This finding indicated that MV/12 and KR/07 infected guinea pigs might have more viral shedding than JS/10 infected guinea pigs. To be noted, the viral RNA of all the three H3N2 CIV strains could be detected in brain tissues in both inoculated mice and guinea pigs, indicating that the H3N2 CIV may have ability to break through the blood-brain barrier.

Similar to the findings in the mouse model, the viral titers of group MV/12 were higher than the titers of groups JS/10 and KR/07 in most tissues regardless of whether the guinea pigs were infected by inoculation or contact. Additionally, the soft palate was the only tissue that the viral titers could be detected in all guinea pigs from the contact group. The viral titers of the soft palate infected by each virus were higher than those of the other tissues except for the lung. Previous studies reported that human influenza virus (A/Changchun/01/2009(H1N1)) could replicate in the lung, trachea, brain and nasal turbinate in guinea pigs [15, 24], and the soft palate is an important adaptation site for transmissible influenza virus [20]. Thus, we speculated that the soft palate might also be a key site for the adaptation of H3N2 CIV. Unlike the BALB/c mouse model, the lung tissues of the guinea pigs in both the inoculated and contact groups all showed obvious gross lesions and corresponding histopathological findings. This result may suggest that the guinea pig was a better host model to evaluate the pathogenicity of H3N2 CIV. Taken together, the results obtained in guinea pigs also demonstrated that the pathogenicity of MV/12 was higher than that of JS/10 and KR/07 and that JS/10 had much wider organ tropism than KR/07. These results were consistent with previous studies that evaluated the pathogenicity of JS/10 [12] and KR/07 [3] using beagle dogs. The gross lesions of beagle dogs infected with KR/07 were limited to the lungs [4]; however, most of the tissues from beagle dogs infected with JS/10, including the heart, liver, spleen, lung, kidney and duodenum, showed varying degrees of lesions and high viral RNA loads. Sequence analysis showed a unique two amino acid insertion in the distal end of the NA stalk in JS/10 compared to KR/07 [10]. Interestingly, our previous study [6] demonstrated that the two amino acid insertion in JS/10 increased viral infectivity and led to a higher proportion of detectable viral RNA in mouse tissues. Therefore, the wider organ tropism may be partially attributed to the presence of the two amino acids.

Reassortment and mutations can drive influenza A virus evolution [36]. Recently, some investigators have confirmed that the influenza virus M gene influences viral replication. Ozaki et al. [37] reported that the PB2 and M genes affected H6 influenza virus replication in chickens. Ma et al. [38] reported that the 2009 pandemic influenza H1N1 virus will facilitate efficient replication and transmissibility in pigs when the neuraminidase (NA) and matrix (M) genes cooperated functionally. Additionally, M gene reassortment in H9N2 influenza virus promoted early infection and replication in chickens [39]. In this study, MV/12 and CA/09 demonstrated an early surge in progeny virus production and more severe pathology than JS/10 and KR/07 in both the mouse and guinea pig models. MV/12 was highly identical (above 99%) to JS/10 and KR/07 in nucleotide sequences of the viral RNA segments, except for the M segment that has been identified to be from CA/09 [11]; therefore, we can reasonably speculate that the M gene may contribute to the higher pathogenicity of MV/12.

## Conclusions

We demonstrated that the Chinese CIV JS/10 virus has wider tissue tropism than the Korean CIV KR/07 virus and that the recombinant H3N2 CIV MV/12 virus showed the highest pathogenicity among the three H3N2 CIV strains. The data presented here indicated that the M gene obtained from pH1N1 may contribute to the pathogenicity of recombinant H3N2 CIV MV/12, although more rigorous future studies will be required. This study highlighted the pathogenicity and transmissibility of H3N2 CIV strains, which will be crucial for understanding the evolutionary characteristics of CIVs and preventing the emergence of potential pandemic strains.

## Abbreviations

ANOVA: Analysis of variance
CIV: Canine influenza virus
dpe: days post exposure
dpi: days post-inoculation
EID50/mL: 50% egg infectious dose
HI: Haemagglutination inhibition
IAV: Influenza A virus
NP: Nucleoprotein;
PBS: Phosphate buffered saline
PCR: Polymerase chain reaction
SPF: Specific Pathogen Free
